# Impact of *Phellinus gilvus* mycelia on growth, immunity and fecal microbiota in weaned piglets

**DOI:** 10.7717/peerj.9067

**Published:** 2020-04-28

**Authors:** Yuqing Sun, Shi Zhong, Bo Deng, Qinsheng Jin, Jie Wu, Jinxi Huo, Jianxun Zhu, Cheng Zhang, Yougui Li

**Affiliations:** 1 Sericultural Research Institute, Zhejiang Academy of Agricultural Science, Hangzhou, China; 2 Institute of Animal Husbandry and Veterinary Science, Zhejiang Academy of Agricultural Sciences, Hangzhou, China; 3 Agricultural Technology Extension Service Center of Nanxun District, Huzhou, Zhejiang, China; 4 Hangzhou Zhengxing Animal Husbandry co. LTD, Linan, Zhejiang, China

**Keywords:** Growth promoting, Piglet, *Phellinus gilvus*, Fecal microbiota, Immunological parameters, Antibiotic

## Abstract

**Background:**

Antibiotics are the most commonly used growth-promoting additives in pig feed especially for weaned piglets. But in recent years their use has been restricted because of bacterial resistance. *Phellinus*, a genus of medicinal fungi, is widely used in Asia to treat gastroenteric dysfunction, hemrrhage, and tumors. *Phellinus* is reported to improve body weight on mice with colitis. Therefore, we hypothesize that it could benefit the health and growth of piglets, and could be used as an alternative to antibiotic. Here, the effect of *Phellinus gilvus* mycelia (SH) and antibiotic growth promoter (ATB) were investigated on weaned piglets.

**Methods:**

A total of 72 crossbred piglets were randomly assigned to three dietary treatment groups (*n* = 4 pens per treatment group with six piglets per pen). The control group was fed basal diet; the SH treatment group was fed basal diet containing 5 g/kg SH; the ATB treatment group was feed basal diet containing 75 mg/kg aureomycin and 20 mg/kg kitasamycin. The experiment period was 28 days. Average daily gain (ADG), average daily feed intake (ADFI), and feed intake to gain ratio were calculated. The concentrations of immunoglobulin G (IgG), interleukin-1β (IL-1β), tumor necrosis factor (TNF)-α and myeloperoxidase (MPO) in serum were assessed. Viable plate counts of *Escherichia coli* in feces were measured. Fecal microbiota was analyzed via the 16S rRNA gene sequencing method.

**Results:**

The ADG (1–28 day) of piglets was significantly higher in SH and ATB treatment groups (*P* < 0.05) compared to the control, and the ADG did not show significant difference between SH and ATB treatment groups (*P* > 0.05). Both SH and ATB treatments increased the MPO, IL-1β, and TNF-α levels in serum compared to the control (*P* < 0.05), but the levels in SH group were all significantly higher than in the ATB group (*P* < 0.05). Fecal microbiological analysis showed that viable *E. coli* counts were dramatically decreased by SH and ATB. The 16S rRNA gene sequencing analysis showed that ATB shifted the microbiota structure drastically, and significantly increased the relative abundance of *Prevotella*, *Megasphaera*, and *Faecalibacterium* genera. But SH slightly influenced the microbiota structure, and only increased the relative abundance of *Alloprevotella* genus.

**Conclusion:**

Our work demonstrated that though SH slightly influenced the microbiota structure, it markedly reduced the fecal *E. coli* population, and improved growth and innate immunity in piglets. Our finding suggested that SH could be an alternative to ATB in piglet feed.

## Introduction

Antibiotics have been used as feed additives in livestock and poultry for decades, but their use has been restricted because of the speculated risk of generating antibiotic resistance in pathogenic bacteria, especially since the European Union banned their use in food-producing animals in 2006 (Castanon, 2007). Consequently, many alternatives have been applied to control or prevent clinical diseases and maximize growth performance. For example, traditional Chinese medicines, which have been widely used in China to treat various diseases and conditions, became important sources of functional feed additives (Liu, Tong & Zhou, 2011). Many Chinese medicines are demonstrated to be effective in promoting health and growth in livestock (Song et al., 2010; Hong et al., 2012).

The *Phellinus* mushroom, which is called Sanghuang in Chinese, is a genus of basidiomycetous fungi belonging to *Hymenochaetaceae*. *Phellinus spp.* has been widely used in Asian countries to treat gastroenteric dysfunction, hemorrhage, and tumors (Song et al., 2008; Chen et al., 2016). The immunity-modulating, anti-inflammatory, and antitumor effects of *Phellinus spp.* are well documented (Zhou et al., 2014; Suabjakyong et al., 2015; Gao et al., 2017; Wang et al., 2017). In our previous study, the extract of *Phellinus* ameliorated intestinal injury and improved the body weight of mice with colitis (Sun et al., 2018). However, little is known about the effect of *Phellinus* on livestock. Therefore, the present research aimed to investigate whether *Phellinus* could promote growth and health of piglets and be an alternative to antibiotic growth promoter (ATB) in feed.

Gut microbiota plays an important role in the digestion, fermentation, and metabolism of many chemical compounds in food (Feng, Chen & Wang, 2018). The bioactivities of functional foods and drugs, such as cranberry (Anhe et al., 2015), ganoderma (Chang et al., 2015), and metformin (Shin et al., 2014), are also actively mediated by the gut microbiota. Therefore, the gut microbiota is a potential target for nutrition intervention to maintain animal health.

In this study, the effect of *Phellinus gilvus* mycelia (SH) on the growth parameters and immunological parameters of weaned piglets was compared with that of ATB. The effects of SH and ATB on the fecal microbiota were also investigated. Our work demonstrated that SH was comparable to ATB in promoting growth of piglets, and it was more effective than ATB in enhancing innate immunity. SH markedly decreased fecal viable *Escherichia coli* counts as ATB did. But unlike ATB, which altered microbiota structure greatly, SH slightly influenced the structure of microbiota.

## Materials & Methods

### Preparation of *P. gilvus* mycelia (SH)

*P. gilvus* strain (preserved at the Sericultural Research Institute, Zhejiang Academy of Agricultural Science, Hangzhou, China) was incubated in sterile liquid culture medium (ingredients are provided in Table S1) in a fermentation tank at 25 °C with stirring speed of 100 r/min. The entire culture process was sterile. After 2 weeks, the mycelia of *P. gilvus* growing in the fermentation tank was separated from the liquid culture medium with a filter and dried at 65 °C. The dried mycelia were then ground into fine powder and stored at −20 °C before use. The SH was tested to have 39.8% crude protein, 15.2% crude fiber, 11.1% crude fat, 4.3% ash, 7.5% water-soluble polysaccharide, and 2.5% polyphenol.

### Experimental design

The experiment was conducted in Hangzhou Zhengxing Animal Husbandry co. LTD (Linan, Zhejiang, China), and all the piglets used in this experiment were from this company. The trial was approved by the farm’s veterinary consultant, and all procedures were compliant with Chinese regulations and were approved by the Medical Ethics Committee of Zhejiang Academy of Agricultural Sciences (No. 2017-050).

A total of 72 crossbred piglets [Duroc × (Landrace × Large White), 7.51 ± 0.88 kg BW, 36 barrows and 36 gilts] weaned at 28 d of age were used in a 28-day feeding trial. The piglets were assigned to three dietary treatment groups with four replicate pens per treatment and six piglets (three barrows and three gilts) per pen. Each pen was equipped with a self-feeder and a nipple waterer. Piglets had free access to water and diets.  A combination of daylight and artificial light was used, and room temperature was controlled at 24−26 °C. The treatments included basal diet without antibiotics or SH (C group), basal diet with 5 g/kg SH (SH group), and basal diet with antibiotics (ATB group). The antibiotic contents were 75 mg/kg aureomycin and 20 mg/kg kitasamycin. The composition and nutrient levels of the basal diet are shown in Table 1. On day 28, blood of eight pigs per treatment (two pigs per pen) was collected from the anterior vena cava using disposable syringes. Rectal fecal samples of ten pigs per treatment (two or three pigs per pen) were obtained. The sampling progress was performed by an experienced veterinary assistant, and no analgesia or anesthetic was given. None of the piglet died all through the experiment period. After the experiment was over, the piglets continued to be kept by Hangzhou Zhengxing Animal Husbandry co. LTD till they were marketed. Fecal samples were divided immediately into two aliquots; one aliquot was stored at −80 °C for microbial 16S rDNA sequencing analysis, and the other aliquot was stored on ice for plate count analysis of *E. coli*.

**Table 1:** Composition and nutritional characteristics of the basal diet.

Items	Content
Ingredients (g/kg of diet)	
Corn	510
Extruded soybean	130
Soybean meal	180
Whey powder	100
Fish meal	50
Monocalcium phosphate	8
Limestone	5
Salt	3
Choline chloride	1
L-lysine⋅HCl	2
DL-methionine	0.8
L-threonine	0.8
Vitamin and medicine Premix^a^	4.4
Trace mineral Premix^b^	5
Analytical composition (g/kg of DM)	
Crude protein	200
Digestible energy (MJ/kg)	142
Crude fiber	28.3
Crude fat	49.6
Digestible lysine	11.4
Digestible methionine	3.3
Digestible threonine	7.4
Digestible tryptophan	2.2
Calcium	7.5
Total phosphorus	6.6

**Notes.**

a Provided per kg of complete diet: 7,500 IU vitamin A; 750 IU vitamin D_3_; 25 IU vitamin E; 2.0 mg vitamin K_3_; 1.875 mg vitamin B_1_; 3.75 mg vitamin B_2_; 2.19 mg vitamin B_6_; 0.025 mg vitamin B_12_; 25 mg niacin; 15.6 mg D-pantothenic acid; 2.0 mg folic acid; 0.1875 mg biotin; 100 g phytase.

b Provided per kg of complete diet: 25 mg Cu (as CuSO_4_⋅5H_2_O); 100 mg Zn (as ZnSO_4_); 30 mg Mn (as MnO_2_); 100 mg Fe (as FeSO_4_⋅H_2_O); 0.3 mg Se (as Na_2_SeO_3_⋅5H_2_O); 0.4 mg I (as Ca(IO_3_)_2_).

### Growth performance measurement

The piglets were individually weighed on days 0, 14, and 28 of the experiment. Feed intake per pen was documented weekly, and the residual feed was measured at the end of the experiment. Average daily gain (ADG), average daily feed intake (ADFI), and feed intake to gain ratio (F/G) were calculated based on these parameters (*n* = 4, a pen was considered as an experimental unit).

### Assay of immunological parameters in serum

Blood samples (*n* = 8 per group, two pigs per pen) were centrifuged at speed of 3,500 rpm for 10 min to obtain serum. The concentrations of immunoglobulin G (IgG), interleukin-1β (IL-1β), tumor necrosis factor (TNF)-α and myeloperoxidase (MPO) in serum were assessed using commercially available enzyme-linked immunosorbent assay (ELISA) kits (R&D Systems, Minneapolis, MN, USA) according to the manufacturer’s instructions. The colorimetric reaction was read in an automated ELISA microplate reader (Versamax, Molecular Devices).

### Viable plate counts of *E. coli*

Rectal fecal samples of ten pigs per treatment (two or three pigs per pen) were obtained and feces from two or three pigs in the same pen were mixed into one sample. The mixed fecal samples (*n* = 4 per group) were serially diluted (10^−1^–10^−7^) in sterilized saline solution. An aliquot of 100 µL of the sample was plated onto MacConkey agar plates and spread evenly by using an L-shaped spreader. Three parallel plates were prepared for each sample dilution. The plates were then inverted and incubated at 37 °C for 24 h. The number of colonies in the plates with 30–300 available isolated colonies was used to calculate *E. coli* population in feces. The calculated equation is as follows:

Viable *E. coli* population in feces, colony-forming unit (CFU)/g = Number of *E. coli* colonies on the plate × dilution ratio of fecal sample/fecal weight.

### Microbiota analysis of fecal samples via 16S rRNA gene sequencing

Microbial genome DNA of fecal samples (*n* = 10 per group, two or three pigs per pen) was isolated and purified using the QIAamp DNA Stool Mini Kit (Qiagen, GmbH Hilden, Germany) in accordance with the manufacturer’s instructions. The V3-V4 hypervariable regions of the 16S rRNA genes were PCR amplified from the microbial genomic DNA by using primers B341F–B785R (B341F: CCTACGGGNGGCWGCAG, B785R: GACTACHVGGGTATCTAATCC). PCR was performed in 25 µL of the reaction mixture with 12.5 µL of 2 × KAPA HiFi HotStart Ready Mix (KAPA Biosystems), 0.25 µL of 25 µM forward and reverse primers, and approximately 10 ng of template DNA. Thermal cycling involved initial denaturation at 95 °C for 3 min, followed by 25 cycles of denaturation at 95 °C for 30 s, annealing at 55 °C for 30 s, and elongation at 72 °C for 30 s and 72 °C for 5 min. The resulting amplicons were gel purified, quantified, pooled, and sequenced. Sequencing libraries were generated using a NEB Next® UltraTM DNA Library Prep Kit for Illumina (NEB, USA) in accordance with the manufacturer’s recommendations. The quality of the library was assessed with a Qubit@ 2.0 Fluorometer (Thermo Scientific) and an Agilent Bioanalyzer 2100 System (Agilent Technologies, Inc.). The library was sequenced on an Illumina Miseq platform, and 2 × 300 bp paired end reads were generated.

Raw sequence reads were filtered and assembled. Sequences with contaminated adapters, undetermined nucleotide, or low complexity were removed. The resulting sequences were clustered into operational taxonomic units (OTUs) by using USEARCH drive 7 at 97% sequence similarity. Chimeric OTUs were removed with UCHIME v4.2. Representative sequences for each OTU were picked and aligned using QIIME 1.8. The Ribosomal Database Project Classifier v2.2 was used to assign a taxonomic rank to each sequence in the representative set. The relative abundance of each OTU was examined at different taxonomic levels. Genetic diversity calculation and taxonomic community assessment were performed with QIIME 1.8 scripts. Sequencing and bioinformatics analyses were conducted by Hangzhou KAITAI Biotechnology Co., Ltd. (Hangzhou, China).

### Statistical analysis

Data were expressed as means or means ± SEM. Significant differences in growth parameters, immunological parameters, viable plate counts of E. coli, and the relative abundance of the genus were analyzed by one-way ANOVA followed by LSD post hoc test by using SPSS 12.0 (Chicago, USA) and the significance was considered at *P* < 0.05. For phylotype analysis, the alpha diversity of the microbiome was calculated on the basis of the OTU level by using USEARCH 7.0. Principal coordinate analysis (PCoA) was performed using R and visualized with the R package ggplot2, and significant differences were evaluated by Adonis analysis (R package vegan) at *P* < 0.05. Comparison of OTU levels between groups were assessed using the edgeR test in R by using the Benjamini and Hochberg false discovery rate, and the significance was considered at *P* < 0.05 and FDR < 0.2.

## Results

### Growth parameters

The ADG of the ATB group and SH group in the entire stage (1–28 days) increased significantly (*P* < 0.05) compared with that of the control group (Table 2). SH treatment increased the ADG greatly at the later stage (15–28 days). The ADFI and F/G throughout the entire experimental period were not significantly changed in ATB or SH group compared with those in control group (*P* > 0.05).

**Table 2:** Effect of *Phellinus gilvus* mycelium (SH) and antibiotic growth promoter (ATB) on the growth parameters in piglets.

Item		Treatment			SEM	*P*-Value
		C	ATB	SH		
ADG	1–14 days	191 ± 12.31	228 ± 7.64	207 ± 9.09	6.85	0.077
	15–28 days	207 ± 16.78^a^	252 ± 18.89^a^^b^	277 ± 10.51^b^	12.0	0.033
	1–28 days	199 ± 9.03^a^	240 ± 8.02^b^	242 ± 9.79^b^	7.57	0.013
ADFI	1–14 days	309 ± 7.07	305 ± 20.90	315 ± 15.31	8.18	0.913
	15–28 days	463 ± 34.55	556 ± 9.21	517 ± 23.75	17.24	0.076
	1–28 days	386 ± 18.95	430 ± 14.09	416 ± 19.23	10.73	0.246
F/G	1–14 days	1.63 ± 0.12	1.34 ± 0.09	1.52 ± 0.06	0.060	0.127
	15–28 days	2.27 ± 0.16	2.23 ± 0.12	1.87 ± 0.06	0.084	0.088
	1–28 days	1.95 ± 0.09	1.80 ± 0.02	1.72 ± 0.06	0.045	0.100

**Notes.**

ADG average daily gain ADFI average daily feed intake F/G feed intake to gain ratio C control

*n* = 4 (a pen was considered as an experimental unit; six pigs per pen); data are shown as means ± SEM.

a,b values with different superscripts in the same row differ significantly (*P* < 0.05).

### Immunological parameters

**Figure 1:**
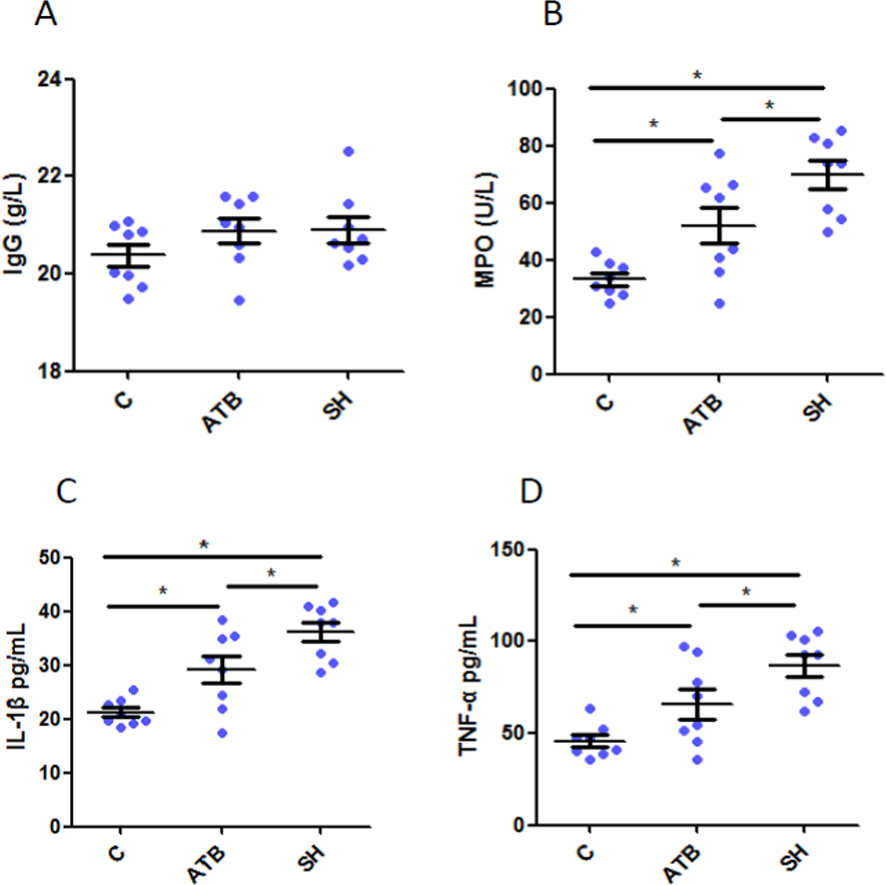
Serum immunological parameters in different treatment groups. (A) Immunoglobulin G (IgG); (B) myeloperoxidase (MPO); (C) interleukin 1 beta (IL-1β); (D) tumor necroses factor alpha (TNF-α). *n* = 8. Data are shown as means ± SEM. *P < 0.05.

We quantified the IgG, the important antibody in adaptive immunity, TNF-α and IL-1β, cytokines secreted mainly by monocyte-macrophages, and MPO, one biomarker of active neutrophil in serum. Results (Fig. 1A) show that IgG levels did not varied among groups, suggesting that ATB and SH did not affected the adaptive immunity. However, the levels of TNF- α, IL-1β and MPO were both increased by treatment of ATB or SH (Figs. 1B–1D), and these cytokine levels in SH group were significant higher than in ATB group (*P* < 0.05). These results indicated the better innate immune response of pigs in SH group than that of the control group and ATB group.

### Viable *E. coli* counts in feces

**Figure 2:**
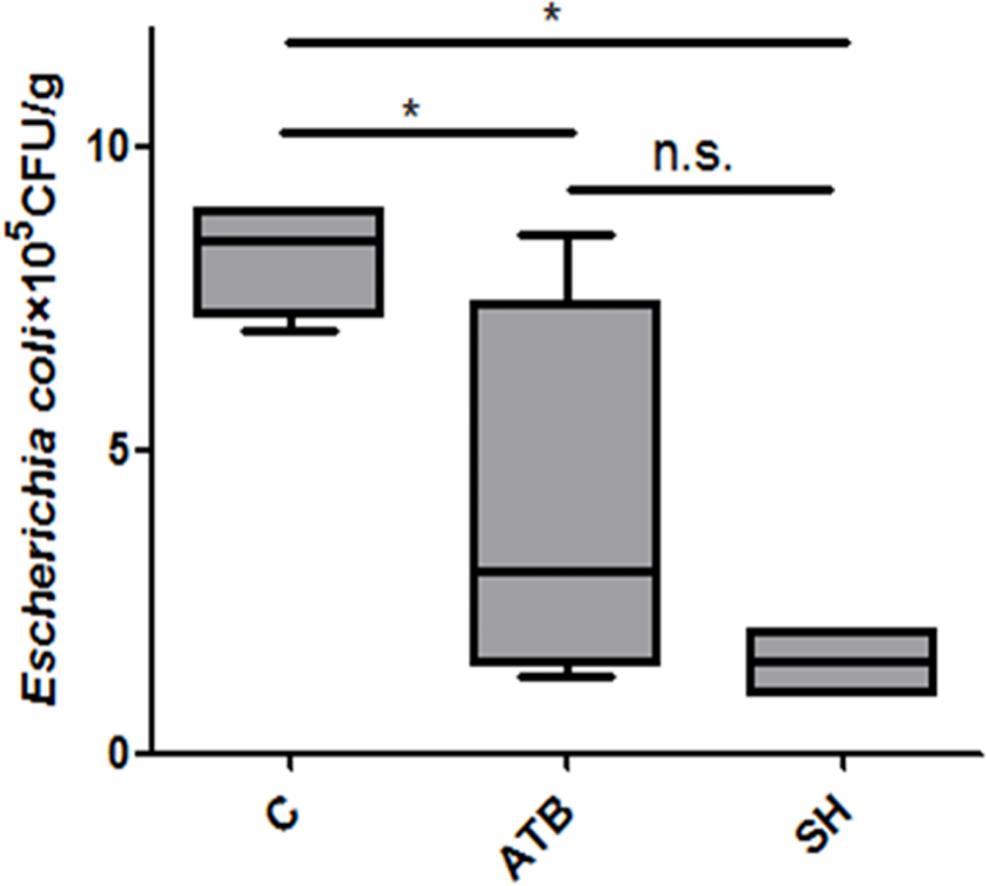
Effect of *Phellinus gilvus.* mycelium (SH) and antibiotic growth promoter (ATB) on viable *Escherichia coli* counts in piglets’feces. *n* = 4; *P < 0.05; n.s., no significant difference (*P* > 0.05).

*E. coli* is a group opportunistic pathogens that may cause diarrhea in piglets. Figure 2 shows that both ATB and SH treatments decreased the viable *E. coli* counts in feces.

### Fecal microbiota structure analysis

**Figure 3:**
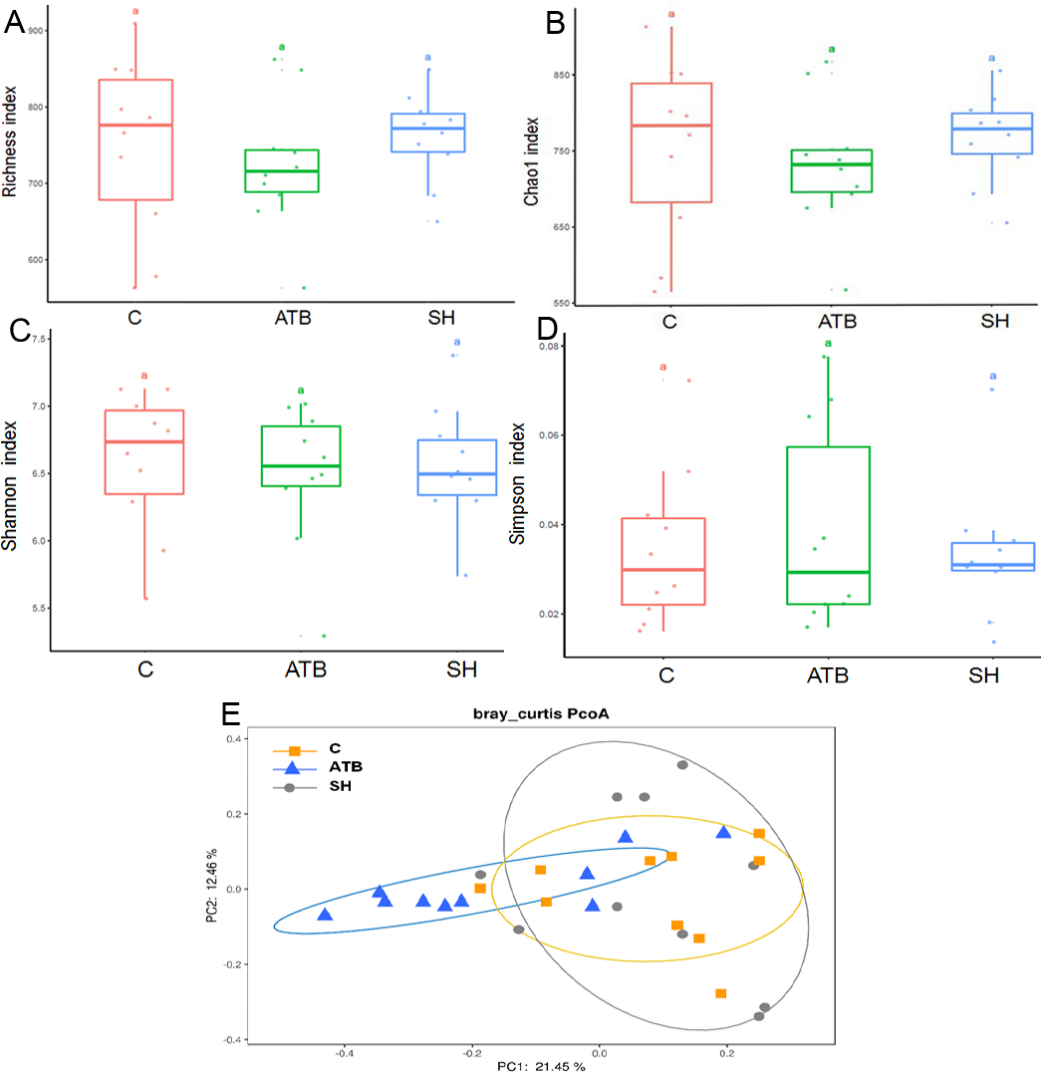
The diversity and structure of the gut microbiota in different groups. (A–D) Diversity as estimated by the Richness, Chao 1, Shannon, and Simpson indices; (E) Principal coordinate analysis (PCoA) plots of microbial communities; *n* = 10.

To further explore the influence of SH and ATB on the gut microbiota, we analyzed the structure of the fecal microbiota by 16S rRNA pyrosequencing. The diversity of the fecal microbes as displayed by the Richness, Chao1, Shannon, and Simpson indices (Figs. 3A–3D) was not significantly changed by SH or ATB treatment (*P* > 0.05). As displayed by PCoA on the OTU level (Fig. 3E), the microbiota of the SH group largely overlapped with the control. The ATB group was distinct from the control group in terms of position (*P* < 0.05), suggesting the significant ATB-induced change in the gut microbiota structure.

**Figure 4:**
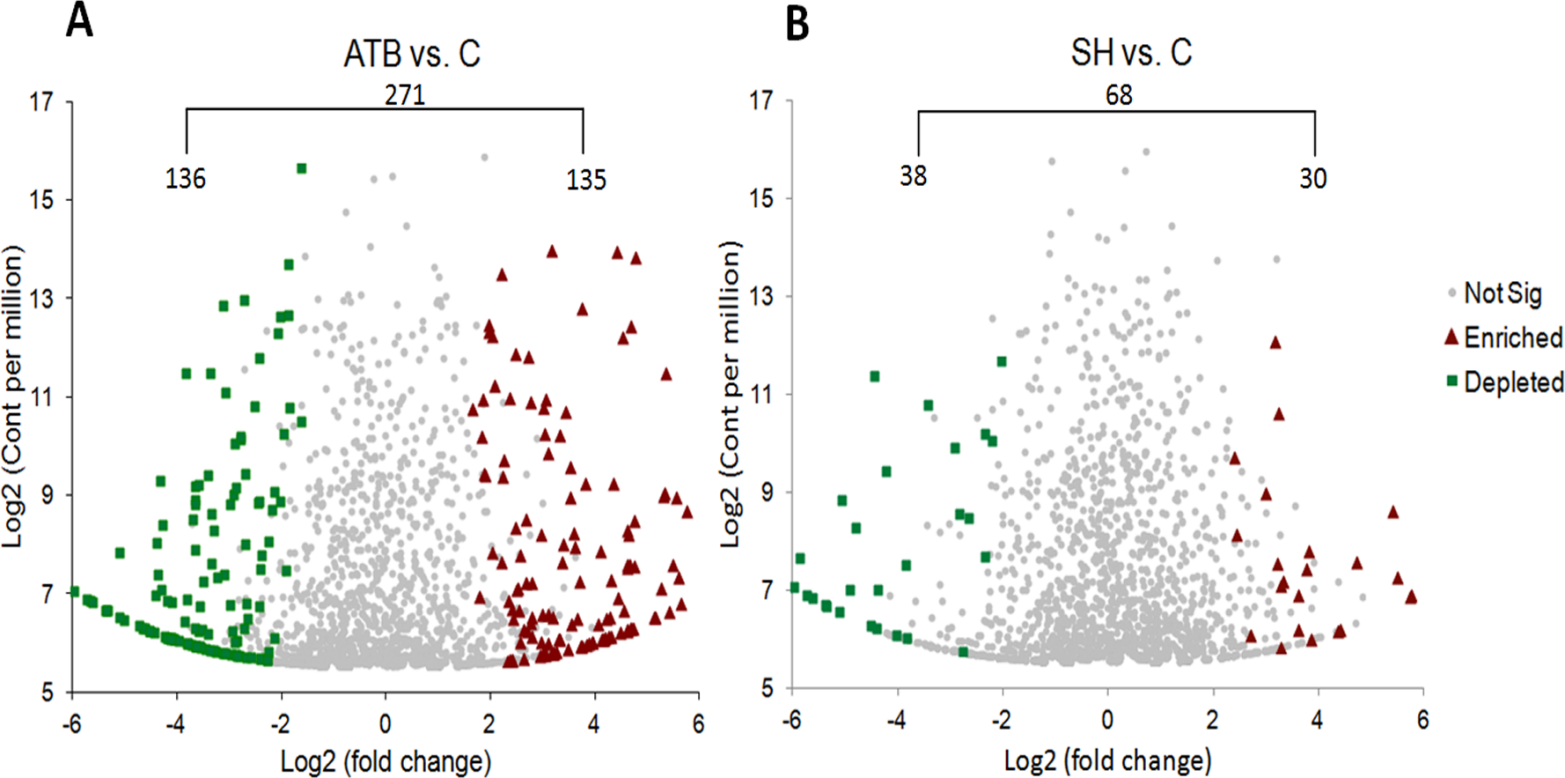
Comparison of OTUsof piglets’ fecal microbiota sequences between control group and treatment groups. (A) ATB group vs. Control group, (B) SH group vs. Control group.

We also compared the relative abundance of each OTU in the SH and ATB groups with those in control group (Fig. 4). It shows that there were 271 OTUs significantly different in relative abundance between ATB group and control group; of these OTUs, 135 OTUs were significantly higher in relative abundance in ATB group than the control, and 136 OTUs were significantly lower in relative abundance in ATB group compared with the control. There were 68 OTUs significantly different in relative abundance between SH group and control group; of these OTUs, 30 OTUs were significantly higher in relative abundance in SH group than the control group, and 38 OTUs were significantly lower in relative abundance in SH group compared with the control.

**Figure 5:**
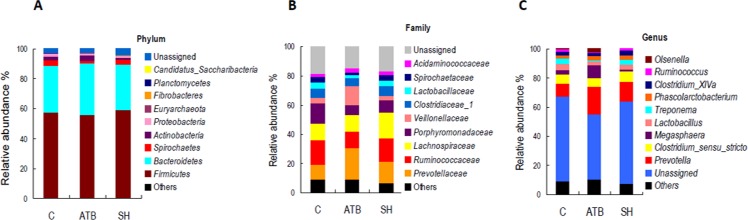
Relative abundance of the top 10 phyla, families, and genera of fecal microbiota in different groups. (A) Accumulated abundance of the top 10 phyla; (B) accumulated abundance of the top 10 families; (C) accumulated abundance of the top 10 genera.

**Figure 6:**
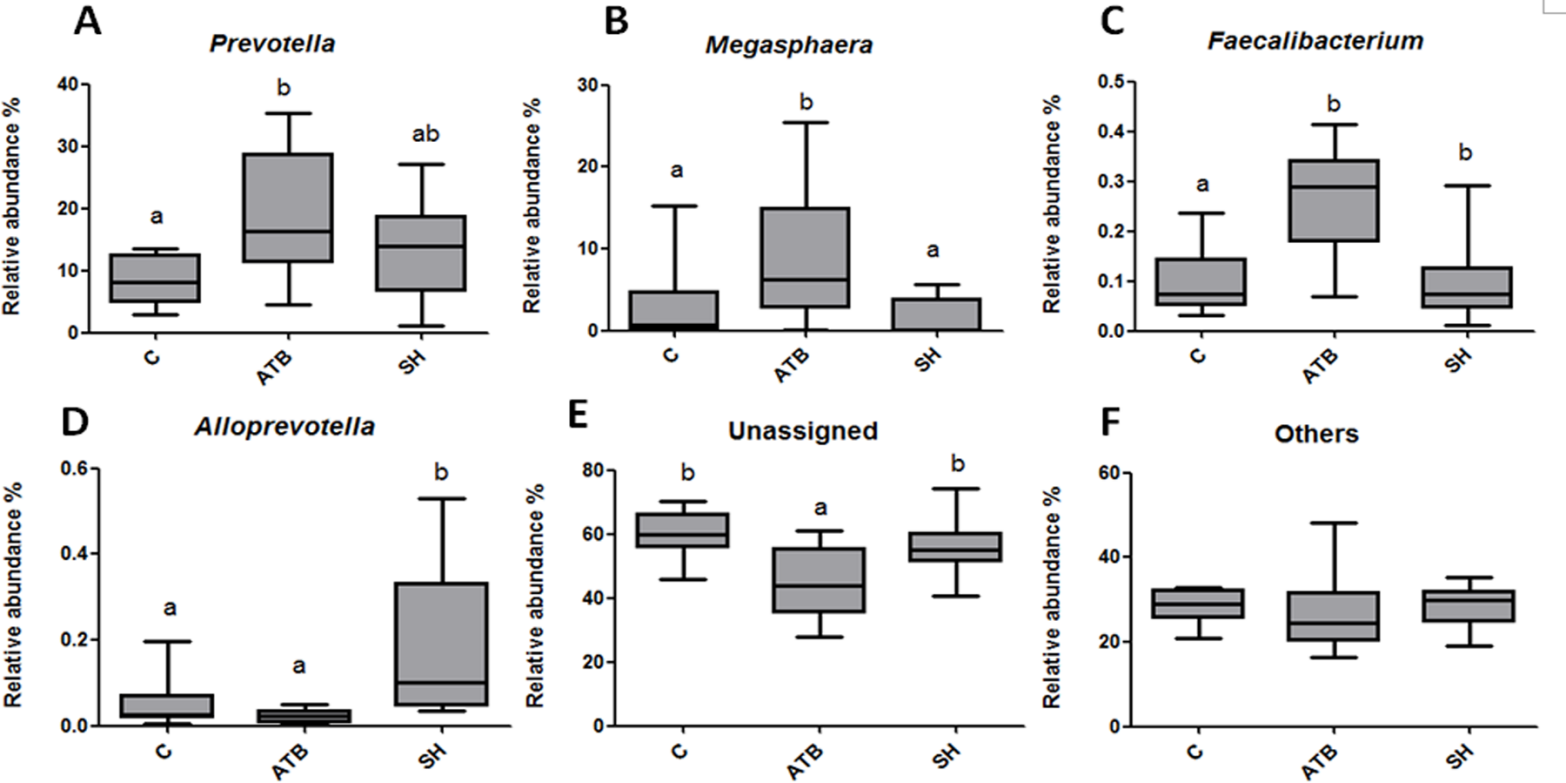
Relative abundance of the genera significantly changedby SH or ATB. (A) *Prevotella*; (B) *Megasphaera*; (C) *Faecalibacterium*; (D) *Alloprevotella*; (E) unassigned genera; (F) other genera. *n* = 10. Data are shown as means means ± SEM. ^*a*,*b*^ different superscripts on bars mean significant difference between groups (*P* < 0.05).

Figure 5 displays the changes in the relative abundance of the top 10 phyla, families, and genera. SH treatment slightly influenced the top 10 phyla, families, and genera compared with the control group. By contrast, ATB remarkably changed the structures of families and genera compared with the control group. Figure 6 shows the genera that were significantly altered in relative abundances by SH or ATB treatment compared with the control. SH treatment increased the relative abundance of *Alloprevotella* (*P* < 0.05). ATB treatment increased the abundance of *Prevotella*, *Megasphaera* and *Faecalibacterium* significantly (*P* < 0.05).

## Discussion

As a result of the restriction of antibiotic growth promoters in feed, search for alternatives to antibiotics has become a hotspot. In the last decade, there has been a noticeable growth in interest in the potential of mushrooms and their application in animal feed. The growth promoting effect has been demonstrated in many mushroom strains in *Basidiomycota* taxon such as *Agaricus bisporus*, *Hericium caput-medusae* (Bull.:Fr.) Pers and *Lentinus edodes* (Giannenas et al., 2010; Shang et al., 2015; Guo et al., 2004). It is reported that dietary inclusion of *Agaricus bisporus* mushroom at 20 g/kg improved growth performance, feed efficiency in broiler chickens (Giannenas et al., 2010). *Lentinus edodes* extract at dose of 5g/kg in feed showed similar efficiency to antibiotic apramycin in improving body weight gain of broiler chickens infected with avian *Mycoplasma gallisepticum* (Guo et al., 2004)*.* Mushroom *Phellinus* is also a genus belonged to *Basidiomycota*. Although multiple pharmacological activities of *Phellinus spp.* have been reported in laboratory animals (Chen et al., 2016; Sun et al., 2018; Wang et al., 2019a; Wang et al., 2019b), its effect on livestock has not been reported yet. In the present study, dietary inclusion of 5 g/kg SH for 28 days significantly increased the ADG (1–28 days) of weaned piglets, and the efficiency was similar to ATB. This result indicated the potent growth promoting effect of SH. Moreover, SH was more potent than ATB in increasing serum IL-1β and TNF-α, cytokines that are mainly secreted by monocyte-macrophages, and MPO, a heme-containing peroxidase plays important role in microbial killing in neutrophils. But the IgG level in serum was not affected by any treatment. Monocyte-macrophages and neutrophils are powerful effector cells of innate immune system. Their microbicidal capacity, phagocytosis, and secretion of cytokines such as IL-1β and TNF-α are enhanced when activated (Sun et al., 2017; Aratani, 2018). IgG, antibodies created and released by plasma B cell, are the most common type of antibody found in blood circulation which participate predominantly in the adaptive immune response (Gudelj, Lauc & Pezer, 2018). Our results indicated that SH had better stimulation effect on innate immunity than the adaptive immunity. The immunomodulation effect is frequently reported in mushrooms, and polysaccharides are deemed to be the major active compounds. Li et al. (2015) found that *Ganoderma-lucidum* polysaccharides significantly increased serum concentrations of IgG and IL-2 in finisher pigs. Dalloul et al. (2006) also reported that mushroom and mushroom-derived lectin enhance innate immunity in broiler chicken. ATB also increased MPO, IL-1β and TNF-α in our study. It was reported that the effects on immunity varied between different ATBs (Cheng et al., 2018). In the light of the different antimicrobial spectrums and immunomodulatory capacities of different antibiotics, it is possible that the immunomodulation of ATB is related to the modulation of specific bacteria. Bacterial metabolites and components such as short chain fatty acids (SCFA) and lipopolysaccharides have been proved to be effective immunomodulatory compounds (Inan et al., 2000; Bäckhed et al., 2001). For SH, the immunomodulatory effect might not fully depend on the microbiota, but also on some components such as polysaccharides, because polysaccharides in *Phellinus spp.* has been proved to directly activate immune cells *in vitro* (Suabjakyong et al., 2015; Liu et al., 2016; Wang et al., 2019a; Wang et al., 2019b).

The gut microbiota is a complex community comprising more than 100 trillion microbes that reside in the gut of humans and animals, and it is demonstrated to be important for the health of their host (Bridgewater et al., 2017; Li et al., 2019; Sircana et al., 2019). Mushrooms are reported to have impact on gut microbiota. In research of Shang et al. (2015), the polysaccharides of *Hericium caput-medusae* (Bull.:Fr.) Pers (HFCP) increased the caecum lactobacilli count, bifidobacteria count, and propionic acid concentration in broiler chickens but decreased the caecum *E. coli* count linearly and quadratically as the HFCP levels increased. Guo et al. (2004) reported that *Lentinus edodes* extract and antibiotic apramycin showed different effects on cecal microbial populations: the antibiotic decreased the cecal bifidobacteria and lactobacilli counts, but *Lentinus edodes* extract increased them. Guo et al. (2004) also found that the higher doses of *Lentinus edodes* extract the chicken given, the higher body weight gain and total aerobe and anaerobe counts the chicken had. The effects of SH on microbiota was also investigated in this study. Firstly, we tested viable *E. coli* counts in feces. *E. coli* is a group of facultative anaerobes in human and animal gut. Some *E. coli* serotypes, such as *E. coli* O157: H7, cause serious diarrhea in pigs (Xia et al., 2018). Antibiotics are often used to treat *E. coli*-induced diarrhea, and the resistance of *E. coli* to antibiotics is frequently reported (Hammerum et al., 2006). Our results showed that the viable *E. coli* counts in feces were dramatically decreased by SH treatment, which indicated the inhibition effect of SH on *E. coli*. Pervious study (Reis et al., 2014) reported that the methanolic extract of *Phellinus linteus* showed strong antibacterial activity against several common pathogens including *E. coli*. Phellinstatin, one polyphenolic compound in *Phellinus spp.* that can be extracted by alcohols, can strongly inhibit bacterial enoyl-ACP reductase and may be one major compound accounting for the antibacterial activity of *Phellinus spp.* (Cho et al., 2011).

In the 16S rRNA pyrosequencing analysis, SH treatment did not greatly change the diversity of the microbiota. SH also affected less OTUs than ATB, indicating the milder effects of SH on the gut microbiota structure than those of ATB. SH treatment increased the relative abundance of *Alloprevotella* genus, a group of SCFA-producing bacteria, which mainly produces butyric acid and acetic acid by fermentation of dietary fibers or indigestible carbohydrates (Cheng et al., 2019). Many reports have demonstrated that polysaccharides can increase the *Alloprevotella* relative abundant in gut (Chen et al., 2019; Cheng et al., 2019). Fibers and polysaccharides are not hydrolyzed by digestive enzymes or absorbed in the upper part of the gastrointestinal tract. These ingredients therefore enter the large intestine and may serve as substrates for the endogenous colonic bacteria. The SH contains a high proportion of fibers and polysaccharides, and this may play a role in the increasing of *Alloprevotella* relative abundant. It should be noted that the 16S rRNA pyrosequencing analysis only displayed the relative abundant of the total bacteria, not the absolute population of the bacteria. Therefore, it cannot be ruled out the possibilities that SH increased or decreased the population of total bacteria.

ATB treatment increased the abundance of *Prevotella*, *Megasphaera* and *Faecalibacterium* genera. *Prevotella*, *Megasphaera* and *Faecalibacterium* are all common members of the gut microbiota in humans and animals (Kim & Isaacson, 2015; Yue et al., 2019; Nuzum et al., 2020), of which *Prevotella* is a dominant genus with high abundance (Kim & Isaacson, 2015). Although *Prevotella* genus takes up a high proportion in the normal flora, its role in human and animal health is controversial, with both positive and negative associations. Some researchers report that *Prevotella* genus play important roles in the utilization of feed proteins (Xu & Gordon, 2003; Han et al., 2015). While in other reports, many *Prevotella* species are linked to inflammation (Bertelsen, Elborn & Schock, 2019; Fteita et al., 2018). Nevertheless, the consistent finding is that high ratio of species in *Prevotella* have natural antibiotic-resistant genes tetQ and ermF (Arzese, Tomasetig & Botta, 2000; Veloo et al., 2019). Therefore, we speculate that the great increase of *Prevotella* in relative abundance might attribute to their relative insensitivity to antibiotics compared to other bacteria. This sepeculation is consistent with the previous research about carbadox, a broad spectrum antibiotic, in which research carbadox increased the relative abundance of *Prevotella* in swine gut but did not change its absolute abundance (Looft et al., 2014).

## Conclusions

Our work demonstrated for the first time that SH could promote growth and activate innate immunity in piglets. Unlike ATB, which altered the microbiota structure drastically, SH slightly influenced the structure of microbiota but only decreased fecal viable *Escherichia coli* counts. Our finding suggested that SH could be an alternative to ATB in piglet feed.

##  Supplemental Information

10.7717/peerj.9067/supp-1 File S1 The culture media ingredients of * Phellinus gilvus*

10.7717/peerj.9067/supp-2 File S2 Growth parameters and immunological parameters of different groups

10.7717/peerj.9067/supp-3 File S3 Immunological parameters in serum and fecal viable plate counts of E. coli

10.7717/peerj.9067/supp-4 File S4 The diversity of the gut microbiota in different groups as estimated by the Richness, Chao 1, Shannon, and Simpson indices

10.7717/peerj.9067/supp-5 File S5 Raw data for PCoA analysis of microbiota structure

10.7717/peerj.9067/supp-6 File S6 Comparison of OTUs between control group and ATB group

10.7717/peerj.9067/supp-7 File S7 The comparison of relative abundance of OTUs in the SH group with those in control group

10.7717/peerj.9067/supp-8 File S8 Data of relative abundance of phyla, families,and genera of fecal microbiota in different groups

10.7717/peerj.9067/supp-9 File S9 Relative abundance of the genera significantly changedby SH or ATB

10.7717/peerj.9067/supp-10 File S10 The clean data of 16s rDNA sequencing of fecal microbiota in C group

10.7717/peerj.9067/supp-11 File S11 The clean data of 16s rDNA sequencing of fecal microbiota in ATB group

10.7717/peerj.9067/supp-12 File S12 The clean data of 16s rDNA sequencing of fecal microbiota in SH group

## Additional Information and Declarations

### DNA Deposition

The following information was supplied regarding the deposition of DNA sequences: The raw data of 16s rDNA sequences are available at Sequence Read Archive (SRA) in NCBI: Group C, SAMN13885614 to SAMN13885623; Group ATB, SAMN13885624 to SAMN13885633; Group SH are from SAMN13885634 to SAMN13885643.

### Data Availability

The following information was supplied regarding data availability: The raw data is available in the Supplemental Files.

## References

[ref-1] Anhe FF, Roy D, Pilon G, Dudonne S, Matamoros S, Varin TV, Marette A (2015). A polyphenol-rich cranberry extract protects from diet-induced obesity, insulin resistance and intestinal inflammation in association with increased Akkermansia spp. population in the gut microbiota of mice. Gut.

[ref-2] Aratani Y (2018). Myeloperoxidase: its role for host defense, inflammation, and neutrophil function. Archives of Biochemistry and Biophysics.

[ref-3] Arzese AR, Tomasetig L, Botta GA (2000). Detection of tetQ and ermF antibiotic resistance genes in Prevotella and Porphyromonas isolates from clinical specimens and resident microbiota of humans. Journal of Antimicrobial Chemotherapy.

[ref-4] Bäckhed F, Söderhäll M, Ekman P, Normark S, Richterdahlfors A (2001). Induction of innate immune responses by *Escherichia coli* and purified lipopolysaccharide correlate with organ- and cell-specific expression of toll-like receptors within the human urinary tract. Cellular Microbiology.

[ref-5] Bertelsen A, Elborn JS, Schock BC (2019). Infection with *Prevotella* nigrescens induces TLR2 signalling and low levels of p65 mediated inflammation in Cystic Fibrosis bronchial epithelial cells. Journal of Cystic Fibrosis.

[ref-6] Bridgewater LC, Zhang C, Wu Y, Hu W, Zhang Q, Wang J, Zhao L (2017). Gender-based differences in host behavior and gut microbiota composition in response to high fat diet and stress in a mouse model. Scientific Reports.

[ref-7] Castanon JIR (2007). History of the use of antibiotic as growth promoters in European poultry feeds. Poultry Science.

[ref-8] Chang CJ, Lin CS, Lu CC, Martel J, Ko YF, Ojcius DM, Lai HC (2015). Ganoderma lucidum reduces obesity in mice by modulating the composition of the gut microbiota. Nature Communications.

[ref-9] Chen Y, Liu D, Wang D, Lai S, Zhong R, Liu Y, Yang C, Liu B, Sarker MR, Zhao C (2019). Hypoglycemic activity and gut microbiota regulation of a novel polysaccharide from Grifola frondosa in type 2 diabetic mice. Food and Chemical Toxicology.

[ref-10] Chen H, Tian T, Miao H, Zhao YY (2016). Traditional uses, fermentation, phytochemistry and pharmacology of Phellinus linteus: a review. Fitoterapia.

[ref-11] Cheng X, Huang F, Zhang K, Yuan X, Song C (2018). Effects of none-steroidal anti-inflammatory and antibiotic drugs on the oral immune system and oral microbial composition in rats. Biochemical and Biophysical Research Communications.

[ref-12] Cheng Y, Sibusiso L, Hou L, Jiang H, Chen P, Zhang X, Wu M, Tong H (2019). Sargassum fusiforme fucoidan modifies the gut microbiota during alleviation of streptozotocin-induced hyperglycemia in mice. International Journal of Biological Macromolecules.

[ref-13] Cho JY, Kwon YJ, Sohn MJ, Seok SJ, Kim WG (2011). Phellinstatin, a new inhibitor of enoyl-acp reductase produced by the medicinal fungus Phellinus linteus. Bioorganic & Medicinal Chemistry Letters.

[ref-14] Dalloul RA, Lillehoj HS, Lee JS, Lee SH, Chung KS (2006). Immunopotentiating effect of a Fomitella fraxinea-derived lectin on chicken immunity and resistance to coccidiosis. Poultry Science.

[ref-15] Feng Q, Chen WD, Wang YD (2018). Gut microbiota: an integral moderator in health and disease. Frontiers in Microbiology.

[ref-16] Fteita D, Könönen E, Gürsoy M, Ma X, Sintim HO, Gürsoy UK (2018). Quorum sensing molecules regulate epithelial cytokine response and biofilm-related virulence of three Prevotella species. Anaerobe.

[ref-17] Gao W, Wang W, Sun W, Wang M, Zhang N, Yu S (2017). Antitumor and immunomodulating activities of six Phellinus igniarius polysaccharides of different origins. Experimental and Therapeutic Medicine.

[ref-18] Giannenas I, Pappas IS, Mavridis S, Kontopidis G, Skoufos J, Kyriazakis I (2010). Performance and antioxidant status of broiler chickens supplemented with dried mushrooms (*Agaricus bisporus*) in their diet. Poultry Science.

[ref-19] Gudelj I, Lauc G, Pezer M (2018). Immunoglobulin G glycosylation in aging and diseases. Cellular Immunology.

[ref-20] Guo FC, Williams BA, Kwakkel RP, Li HS, Li XP, Luo JY, Li WK, Verstegen MWA (2004). Effects of mushroom and herb polysaccharides, as alternatives for an antibiotic, on the cecal microbial ecosystem in broiler chickens. Poultry Science.

[ref-21] Hammerum AM, Sandvang D, Andersen SR, Seyfarth AM, Porsbo LJ, Frimodt-Moller N, Heuer OE (2006). Detection of sul1, sul2 and sul3 in sulphonamide resistant *Escherichia coli* isolates obtained from healthy humans, pork and pigs in Denmark. International Journal of Food Microbiology.

[ref-22] Han X, Yang Y, Yan H, Wang X, Qu L, Chen Y (2015). Rumen bacterial diversity of 80 to 110-day-old goats using 16s rRNA sequencing. PLOS ONE.

[ref-23] Hong D, Zhong Y, Liu F, Yang K, Yu J, Xu J (2012). Regulating effects and mechanisms of Chinese medicine decoction on growth and gut hormone expression in heat stressed pigs. Livestock Science.

[ref-24] Inan MS, Rasoulpour RJ, Yin L, Hubbard AK, Rosenberg DW, Giardina C (2000). The luminal short-chain fatty acid butyrate modulates NF-kappaB activity in a human colonic epithelial cell line. Gastroenterology.

[ref-25] Kim HB, Isaacson RE (2015). The pig gut microbial diversity: understanding the pig gut microbial ecology through the next generation high throughput sequencing. Veterinary Microbiology.

[ref-26] Li XL, He LP, Yang Y, Liu FJ, Cao Y, Zuo JJ (2015). Effects of extracellular polysaccharides of Ganoderma lucidum supplementation on the growth performance, blood profile, and meat quality in finisher pigs. Livestock Science.

[ref-27] Li YG, Zhong S, Yu JQ, Sun YQ, Zhu JX, Ji DF, Wu CM (2019). The mulberry-derived 1-deoxynojirimycin (DNJ) inhibits high-fat diet (HFD)-induced hypercholesteremia and modulates the gut microbiota in a gender-specific manner. Journal of Functional Foods.

[ref-28] Liu HW, Tong JM, Zhou DW (2011). Utilization of Chinese herbal feed additives in animal production. Agricultural Sciences in China.

[ref-29] Liu MM, Zeng P, Li XT, Shi LG (2016). Antitumor and immunomodulation activities of polysaccharide from *Phellinus baumii*. International Journal of Biological Macromolecules.

[ref-30] Looft T, Allen HK, Casey TA, Alt DP, Stanton TB (2014). Carbadox has both temporary and lasting effects on the swine gut microbiota. Frontiers in Microbiology.

[ref-31] Nuzum ND, Loughman A, Szymlek-Hendy A, Hendy A, Teo WP, Macpherson H (2020). Gut microbiota differences between healthy older adults and individuals with Parkinson’s disease: a systematic review. Neuroscience & Biobehavioral Reviews.

[ref-32] Reis FS, Barreira JCM, Calhelha RC, Van Griensven LJID, Ciric A, Glamoclija J, Sokovic M, Ferreira ICFR (2014). Chemical characterization of the medicinal mushroom Phellinus linteus (Berkeley & Curtis) Teng and contribution of different fractions to its bioactivity. LWT—Food Science and Technology.

[ref-33] Shang HM, Song H, Wang LN, Wu B, Ding GD, Jiang YY, Yao X, Shen SJ (2015). Effects of dietary polysaccharides from the submerged fermentation concentrate of hericium caput-medusae (bull.:fr.) pers. on fat deposition in broilers. Journal of the Science of Food and Agriculture.

[ref-34] Shin NR, Lee JC, Lee HY, Kim MS, Whon TW, Lee MS, Bae JW (2014). An increase in the Akkermansia spp. population induced by metformin treatment improves glucose homeostasis in diet-induced obese mice. Gut.

[ref-35] Sircana A, De Michieli F, Parente R, Framarin L, Leone N, Berrutti M, Musso G (2019). Gut microbiota, hypertension and chronic kidney disease: recent advances. Pharmacological Research.

[ref-36] Song TY, Lin HC, Yang NC, Hu ML (2008). Antiproliferative and antimetastatic effects of the ethanolic extract of Phellinus igniarius (Linnearus: Fries) Quelet. Journal of Ethnopharmacology.

[ref-37] Song X, Xu J, Wang T, Liu F (2010). Traditional Chinese medicine decoction enhances growth performance and intestinal glucose absorption in heat stressed pigs by up-regulating the expressions of SGLT1 and GLUT2 mRNA. Livestock Science.

[ref-38] Suabjakyong P, Nishimura K, Toida T, Van Griensven LJ (2015). Structural characterization and immunomodulatory effects of polysaccharides from Phellinus linteus and Phellinus igniarius on the IL-6/IL-10 cytokine balance of the mouse macrophage cell lines (RAW 264.7). Food & Function.

[ref-39] Sun KY, Xu DH, Xie C, Plummer S, Tang J, Yang XF, Ji XH (2017). Lactobacillus paracasei modulates LPS-induced inflammatory cytokine release by monocyte-macrophages via the up-regulation of negative regulators of NF-kappaB signaling in a TLR2-dependent manner. Cytokine.

[ref-40] Sun YQ, Zhong S, Yu JQ, Zhu JX, Ji DF, Hu GY, Wu CM, Li YG (2018). The aqueous extract of Phellinus igniarius (SH) ameliorates dextran sodium sulfate-induced colitis in C57BL/6 mice. PLOS ONE.

[ref-41] Veloo ACM, Baas WH, Haan FJ, Coco J, Rossen JW (2019). Prevalence of antimicrobial resistance genes in Bacteroides spp. and Prevotella spp. Dutch clinical isolates. Clinical Microbiology and Infection.

[ref-42] Wang FF, Liu F, Shi C, Ma W, Wang KJ, Li N (2017). Cytotoxic Activities of Fractions of the Willow Bracket Medicinal Mushroom, Phellinus igniarius (Agaricomycetes), and the Induction of Cell Cycle Arrest and Apoptosis in MGC-803 Cells. International Journal of Medicinal Mushrooms.

[ref-43] Wang YY, Ma H, Ding ZC, Yang Y, Wang WH, Zhang HN, Yan JK (2019b). Three-phase partitioning for the direct extraction and separation of bioactive exopolysaccharides from the cultured broth of Phellinus baumii. International Journal of Biological Macromolecules.

[ref-44] Wang YQ, Mao JB, Zhou MQ, Jin YW, Lou CH, Dong Y, Shou D, Hu Y, Yang B, Jin CY, Shi HC, Zhao HJ, Wen CP (2019a). Polysaccharide from Phellinus igniarius activates TLR4-mediated signaling pathways in macrophages and shows immune adjuvant activity in mice. International Journal of Biological Macromolecules.

[ref-45] Xia Y, Bin P, Liu S, Chen S, Yin J, Liu G, Tang Z, Ren W (2018). Enterotoxigenic *Escherichia coli* infection promotes apoptosis in piglets. Microbial Pathogenesis.

[ref-46] Xu J, Gordon JI (2003). Honor thy symbionts. Proceedings of the National Academy of Sciences of the United States of America.

[ref-47] Yue SJ, Wang WX, Yu JG, Chen YY, Shi XQ, Yan D, Zhou GS, Zhang L, Wang CY, Duan JA, Tang YP (2019). Gut microbiota modulation with traditional Chinese medicine: a system biology-driven approach. Pharmacological Research.

[ref-48] Zhou C, Jiang SS, Wang CY, Li R, Che HL (2014). Different immunology mechanisms of Phellinus igniarius in inhibiting growth of liver cancer and melanoma cells. Asian Pacific Journal of Cancer Prevention.

