# Supplementary material for: Impact of Phellinus gilvus mycelia on growth, immunity and fecal microbiota in weaned piglets
Source: PeerJ. 2020 Apr 28;8:e9067. doi: 10.7717/peerj.9067 (PMC7194088; doi:10.7717/peerj.9067)
Supplement: File S1 [file peerj-08-9067-s001.doc]

**Impact of *Phellinus gilvus* mycelium on growth,** [**immunity**](javascript:;) **and fecal microbiota in weaned piglets**

Yuqing Sun, Shi Zhong, Bo Deng, Qinsheng Jin, Jie Wu, Jinxi Huo, Jianxun Zhu, Cheng Zhang, Yougui Li

**Supplementary Table S1.** The culture media ingredients of *Phellinus gilvus.*

| Ingredients | Content |
| --- | --- |
| Potato | 200g |
| Sucrose | 20g |
| MgSO_4_ | 1.5g |
| KH_2_PO_4_ | 1.5g |
| Water | 1000mL |

Note: scale up according to this proportion for more culture medium. The culture media was boiled, homogenated and filtered, and the supernatant was used for the culture of *P. gilvus*.
